# Parallel RNAi screens across different cell lines identify generic and cell type-specific regulators of actin organization and cell morphology

**DOI:** 10.1186/gb-2009-10-3-r26

**Published:** 2009-03-05

**Authors:** Tao Liu, David Sims, Buzz Baum

**Affiliations:** 1 MRC Laboratory of Molecular Cell Biology, UCL, Gower Street, London WC1E 6BT, UK; 2 The Institute of Cancer Research, Chester Beatty Laboratories, Fulham Road, London SW3 6JB, UK

## Abstract

**Background:**

In recent years RNAi screening has proven a powerful tool for dissecting gene functions in animal cells in culture. However, to date, most RNAi screens have been performed in a single cell line, and results then extrapolated across cell types and systems.

**Results:**

Here, to dissect generic and cell type-specific mechanisms underlying cell morphology, we have performed identical kinome RNAi screens in six different *Drosophila *cell lines, derived from two distinct tissues of origin. This analysis identified a core set of kinases required for normal cell morphology in all lines tested, together with a number of kinases with cell type-specific functions. Most significantly, the screen identified a role for *minibrain *(*mnb/*DYRK1A), a kinase associated with Down's syndrome, in the regulation of actin-based protrusions in CNS-derived cell lines. This cell type-specific requirement was not due to the peculiarities in the morphology of CNS-derived cells and could not be attributed to differences in *mnb *expression. Instead, it likely reflects differences in gene expression that constitute the cell type-specific functional context in which *mnb*/DYRK1A acts.

**Conclusions:**

Using parallel RNAi screens and gene expression analyses across cell types we have identified generic and cell type-specific regulators of cell morphology, which include *mnb*/DYRK1A in the regulation of protrusion morphology in CNS-derived cell lines. This analysis reveals the importance of using different cell types to gain a thorough understanding of gene function across the genome and, in the case of kinases, the difficulties of using the differential gene expression to predict function.

## Abstract 2

Parallel RNA interference screens and gene expression arrays in six Drosophila cell lines identified regulators of cell morphology, including a neuronal function for the kinase minibrain/DYRK1A in the regulation of protrusion morphology.

## Background

A diversity of cell shapes is a fundamental feature of multicellular life. Cell type-specific forms arise during development as the products of a cell differentiation program that refines patterns of gene expression to yield cells with a form and behavior appropriate to their function. To establish how the forms that characterize cells from different lineages are generated, we have used *Drosophila *cell lines derived from distinct tissues as a model system.

**Figure 1:**
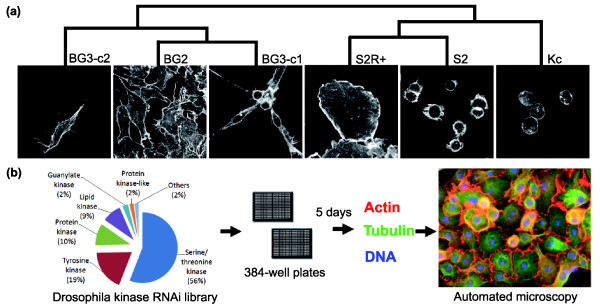
The cell morphology and gene expression profiles of six *Drosophila *cell lines. **(a) **The three CNS-derived cell lines BG2-c2, BG3-c1 and BG3-c2 have a bipolar, spiky cell shape, whereas the three embryonic hemocyte-derived cell lines S2, S2R+ and Kc167 have a symmetrical morphology. Gene expression profiles for each cell line were normalized and hierarchical clustering was used to generate the dendrogram shown. This analysis reveals that cell lines from the same origin have closely related gene expression profiles (Table 1). **(b) **Kinase RNAi screens were carried out in all six cell lines. An RNAi library targeting 265 kinases and kinase regulatory subunits (Additional file 1) was combined with cells in 384-well plates and incubated for 5 days before fixing and staining to visualize F-actin, microtubules and DNA.

*Drosophila *cell lines provide a good model for such an analysis, since multiple cell lines have been derived from diverse tissues, including hemocytes [1-3], neuronal tissue [4] and imaginal discs [5,6], and because the cell lines have morphologies that appear consistent with their lineage. Thus, S2 and S2R+ cells have broad lamelliopodia and are similar in both form and behavior to larval blood cells [6] (D Sims *et al*., unpublished data), while BG1, BG2 and BG3 nervous system-derived cell lines have a common morphology and cyto-architecture [6], which includes filopodia embedded in lamellipodia [7], reminiscent of those seen in some neuronal growth cones [8]. Cell type-specific differences in gene expression are likely to underlie the morphological diversity of cells of different types, leading to differences in the activity of specific signaling pathways and cytoskeletal regulators that control cell form [9]. The genes involved, however, remain largely unknown. In this study, we have used a combination of gene expression microarrays and RNA interference (RNAi) screens to identify cytoskeletal regulators across a panel of *Drosophila *cell lines, enabling us to look for correlations between gene expression and function. Since the structural components of the cytoskeleton and their core regulators (for example, cofilin and profilin) function in a broadly similar way across cell types, we focused our analysis on the kinome to identify cell type-specific differences in the regulation of this basic cytoskeletal machinery. Kinases are a well-defined family of proteins characterized by a common catalytic domain that regulate myriad cellular processes, including the cytoskeleton, and hence cell shape [10-12]. Based on sequence, they can be divided into a number of broad subfamilies with different substrates [13] (Figure 1). To functionally characterize this set of proteins identified by primary sequence, we used genomic sequence information to construct a *Drosophila *kinase RNAi library targeting each gene at least once. In addition, approximately 70% of genes were targeted using two independent double-stranded RNAs (dsRNAs), enabling us to estimate false positive and false negative rates. This RNAi library was then used to screen six different cell lines from two different tissues of origin for novel genes involved in the generation of cell form. In doing so, we identified several common regulators of cell behavior and morphology, together with a set of cell type-specific kinases. Importantly, this analysis revealed that, when considering the kinome, gene expression signatures are a poor measure of cell type-specific differences in gene function.

## Results

### Cell lines from the same origin display similar morphologies and gene expression patterns

*Drosophila *S2, S2R+ (an original isolate of the S2 line [3]) and Kc167 cells, which originate from embryonic hemocytes [1-3], are relatively symmetrical in shape and non-motile (Figure 1a). In addition, these hemocyte-derived cell lines have a propensity to develop lamellipodia rather than filopodia [14]. By contrast, BG2-c2, BG3-c1 and BG3-c2 cells originate from neuronal tissue [4], have a polarized shape characterized by long actin-rich protrusions embedded in lamellipodia [7] (Figure 1a), and are motile (S Bai, B Baum and AJ Ridley, unpublished). BG3-c1 and BG3-c2 represent different clonal isolates from a single primary culture [4]. To determine whether the common origins of these six cell lines are reflected in their respective gene expression profiles, we carried out microarray gene expression analysis on each of the six lines (see Materials and methods). Using hierarchical clustering to analyze these results, it was clear that cell lines from the same tissue of origin have related patterns of gene expression (Figure 1a and Table 1), leading us to conclude that these cell lines are suitable *in vitro *models in which to study the regulatory networks underlying these two distinct cell type-specific morphologies.

**Table 1:** Correlation of microarray and RNAi hit profiles across cell lines

	Kc	S2	S2R+	BG3-c1	BG3-c2	BG2-c2
Kc		**0.36**	**0.25**	0.05	0.16	0.14
S2	***0.56***		**0.44**	0.03	0.21	0.12
S2R+	***0.52***	***0.30***		-0.01	0.08	0.03
BG3-c1	*0.01*	*0.21*	*-0.07*		**0.25**	**0.29**
BG3-c2	*0.00*	*0.17*	*-0.20*	***0.50***		**0.42**
BG2-c2	*-0.09*	*0.07*	*-0.10*	***0.50***	***0.63***	

Corrrelation of microarray and RNAi hit profiles across cell lines. The similarities between microarray gene expression profiles (non-italic, top right section) and RNAi hit profiles (italic, bottom left section) of 6 different cell lines as measured using a Pearson correlation coefficient. Perfect correlation = 1, no correlation = 0. Entries in bold show the highest correlation, indicating that in both the microarray and RNAi data, cells with similar origins are most similar to each other.

### Parallel RNAi screens reveal cell type-specific phenotypes

We have designed and constructed a dsRNA library targeting 265 *Drosophila *kinases and kinase regulatory subunits (Figure 1b; Additional data file 1), which could be used to carry out a comparable functional analysis across cell types. Each kinome screen was carried out in duplicate, in 384-well plates using the bathing method [15] (in the absence of transfection reagent). In each case, following plating, cells were incubated for 5 days to allow for protein turnover, before being fixed and stained to visualize actin filaments, microtubules and DNA. Images were then acquired using an automated microscope (Figure 1b). For each cell line, dsRNAs causing defects in cell morphology were identified by eye and classified using a controlled vocabulary. All cell images and hit annotations are available online through the online FLIGHT database [16].

Genes yielding a similar RNAi phenotype when targeted using multiple non-overlapping dsRNAs are likely to represent true hits, based on the low chances of different dsRNAs sharing the same off-target effects. However, in cases in which one out of two dsRNAs targeting the same transcript(s) elicit a phenotype, one of the two must be either a false positive or a false negative [17]. As with classical genetic screens, our major concern was to identify the false positives amongst this set [17]. False positives can arise as the result of problems with the gene annotation or because of experimental artifacts. In addition, false positives can arise as result of sequence-specific off-target effects, due to short regions of homology to unintended secondary transcripts in long dsRNAs [18], even though the use of long dsRNAs is thought to minimize this problem through the generation of a diverse pool of small interfering RNAs [19]. To estimate false positive and false negative rates in this study, we focused our attention on genes in the screen yielding a phenotype when targeted with one out of two dsRNAs. Each of these genes was then targeted with a third independent dsRNA. This analysis identified 4 false positives out of a total of 22 hits in S2R+ cells, and a single false positive out of 15 hits in the BG2 cell screen (Additional data file 2). Based on these data, we estimate a false positive rate for our experiment of 7-18%, and a false negative rate of 13-27%, depending on the cell line. Importantly, two-thirds of the false negative results could be attributed to defects with the library RNAi plates or dsRNA quality, as assessed by agarose gel electrophoresis during library construction (Additional data file 2).

**Figure 2:**
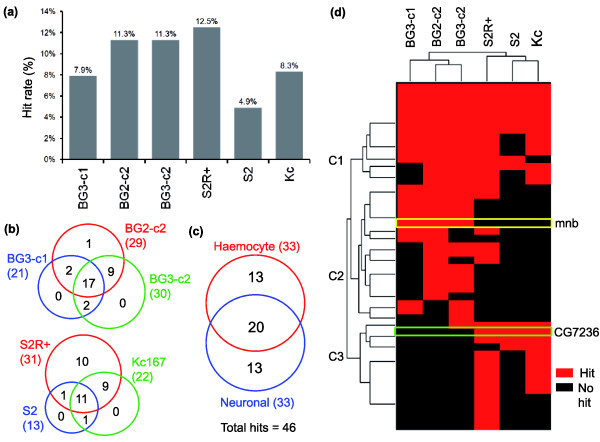
Parallel RNAi screens reveal cell line-specific phenotypes. **(a) **Different cell lines exhibited different hit rates in RNAi screens (Additional file 3). **(b) **Venn diagrams depict the segregation of screen hits between related cell lines. **(c) **A Venn diagram depicts the classification of hits into three distinct classes: those that are hits in both CNS and hemocyte cell lines; those that are hits in neuronal cell lines only; and those that are hits in hemocyte cell lines only. **(d) **Hierarchical clustering of hits across cell lines (depicted in the form of a tree) was used to give a more detailed picture of the three hit classes. Two hits of particular interest, *CG7236 *and *minibrain *(*mnb*), are highlighted. Note that the relationships defined by the functional analysis (depicted in the form of a tree at the top of figure) mirror the relationships defined by the microarray analysis (see Table 1 for the Pearson correlation coefficients in each case).

After elimination of false positives, 17.3% (46 out of 265) of the kinases screened yielded a visible phenotype in at least one of six cell lines (Additional data file 3). This hit rate was similar to that determined in a related screen [14], but varied considerably across lines (Figure 2a; Additional data file 3). Much of the variation in hit rates across cell lines is likely to reflect variation in the ease of identifying defects in cell morphology in each line, since all the phenotypes identified in the BG3-c1 cell line, which is prone to grow in clumps, were also seen in at least one of the better spread central nervous system (CNS) lines (Figure 2b). Similarly, there were only two genes that yielded an RNAi phenotype in S2 or Kc167 cells that did not show up as a hit in the screen in large, well-spread S2R+ cells (Figure 2b). By contrast, there were significant differences in the kinase requirements of hemocyte and CNS-derived lines (Figure 2c,d) as expected based on the differences in the form and gene expression profiles that separate these two sets of lines (Figure 1a). This indicates that both gene expression and function can be used as indicators of a common origin. Using these data, it was possible to identify a set of cell type-specific hits (Figure 2d). However, there was no detectable bias in the number of hits in each kinase class between the two different tissues of origin (Table 2).

**Table 2:** Breakdown of RNAi screen hits according to kinase families

	Total	Neuronal	%	Hemocyte	%
Serine/threonine kinase	146	20	13.7	22	15.1
Tyrosine kinase	49	5	10.2	6	12.2
Protein kinase	26	3	11.5	1	3.8
Lipid kinase	23	3	13.0	2	8.7
Guanylate kinase	7	0	0.0	1	14.3
Protein kinase-like	5	0	0.0	0	0.0
Others	5	2	40.0	1	20.0

Since our goal was the identification of cell type-specific differences in the regulation of cell morphology, we clustered hits across all six cell lines. This revealed three strong clusters (Figure 2d). The first (C1) contains genes that have a strong phenotype in almost all cell lines tested, and is enriched in genes that participate in fundamental cell biological processes such as cell cycle control (for example, *cdc2*, *polo *and *ial*). The second cluster (C2) contains genes that elicit a phenotype in cell lines of CNS origin, and the third cluster (C3) identified hits specific to hemocyte-derived cell lines. We focused our subsequent analysis on genes with morphological phenotypes specific to one tissue type of origin.

### *CG7236 *displays a hemocyte-specific phenotype

**Figure 3:**
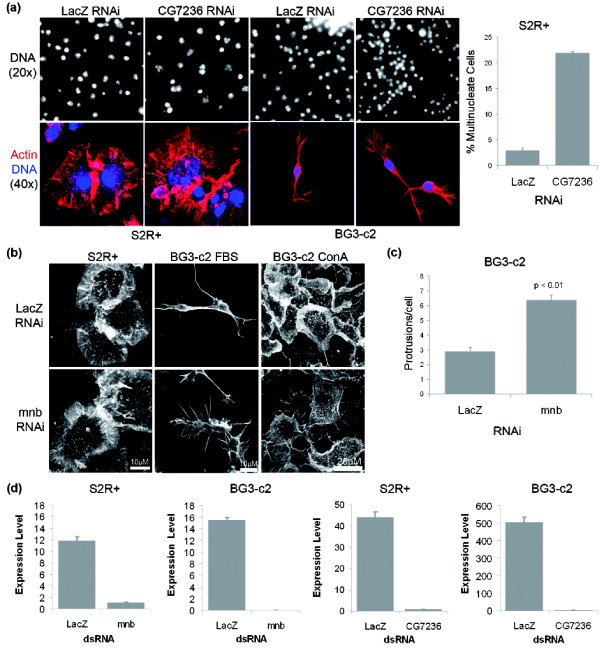
*CG7236 *and *minibrain *show cell line-specific phenotypes. **(a) **Silencing of the cdc2-related kinase *CG7236 *in S2R+ cells gives rise to large cells that frequently contain multiple nuclei or a single large nucleus, whereas silencing in BG3-c2 cells has no discernable phenotype. **(b) **Silencing of the DYRK family kinase *minibrain *in BG3-c2 cells causes an increase in peripheral actin and an increase in the number of protrusions per cell, whereas silencing in S2R+ cells has no phenotype. Also, the BG3-c2 cells forced to spread by plating on concanavalin A (ConA) exhibit large lamellipodia when in the presence of a non-targeting dsRNA, but not in the presence of *mnb *dsRNA. **(c) **Quantification of the *mnb *RNAi phenotype shows a significant twofold increase in the number of long finger-like protrusions around the cell body. **(d) **Q-PCR analysis reveals that *CG7236 *and *minibrain *are effectively silenced by RNAi reagents in both S2R+ and BG3-c2 cells. Error bars indicate the standard error of the mean.

The C3 cluster identified a cyclin-dependent kinase, *CG7236 *[20], which elicited an RNAi phenotype only when targeted in hemocyte cell lines. Cyclin-dependent kinases are known to regulate cell cycle-dependent changes in cell organization together with a host of other processes, such as RNA Polymerase II activity [21]. In hemocyte cell lines RNAi-mediated silencing of *CG7236 *led to the accumulation of large cells with multiple or enlarged nuclei (Figure 3a), as verified using independent dsRNAs and confocal imaging (Figure 3a, bottom panels). This suggests a role for *CG7236 *in the regulation of the cell division cycle. However, RNAi-mediated silencing of *CG7236 *caused no detectable change in the appearance of neuronal cell lines such as BG3-c2 (Figure 3a), even though a quantitative PCR (Q-PCR) analysis revealed that *CG7236 *is both expressed and effectively silenced by RNAi in both S2R+ and BG3-c2 cells (Figure 3d). *CG7236 *has not been studied in detail before, but was previously identified as a cell cycle kinase in an RNAi screen in S2 cells [22], and as having a cytokinesis defect in RNAi screens in *Drosophila *hemocyte cell lines [14,23,24]. By analyzing its function across cell types, our analysis suggests that *CG7236 *differs from many other kinases involved in cell cycle control in performing a cell type-specific function.

### *Mnb *modulates actin-based protrusions in CNS-derived celllines

The C2 cluster identified *minibrain *(*mnb*) as a gene that has a strong morphological RNAi phenotype in all neuronal cell lines tested, without eliciting a visible RNAi phenotype in hemocyte cell lines (Figure 3b; Additional data file 4). As before, the specificity of the RNAi phenotype was confirmed using two sequence-independent dsRNAs in BG3-c2 cells to minimize the chances of sequence-specific off-target effects [25]. *mnb *encodes an evolutionarily conserved member of the DYRK (dual specificity tyrosine-phosphorylation-regulated kinase) family of serine/threonine protein kinases [26]. It was first identified in *Drosophila *as a gene involved in post-embryonic neurogenesis, since all strong loss-of-function *mnb *mutants generate animals with behavioral defects and adult flies with a specific and marked reduction in the size of optic lobes and central brain hemispheres [27]. Furthermore, DYRK1A, a human homolog of *mnb*, has been mapped within the Down's syndrome critical region of chromosome 21 and is over-expressed in Down's syndrome embryonic brain [28]. These data support a specific role for *mnb *in the regulation of neuronal cell morphology.

The *mnb *phenotype was similar across all three neuronal cell lines tested (Figure 3b; Additional data file 4), with silencing of *mnb *expression leading to a significant (>2-fold) increase in the number of long finger-like protrusions around the cell body (quantified in Figure 3c), and an increase in cortical F-actin levels, and reduced cell numbers (Additional data file 5). Significantly, such filopodia are absent from hemocyte-derived cell lines, but are seen in all CNS-derived *Drosophila *cell lines tested [4,7,29]. Moreover, they are superficially similar to actin-based protrusive structures seen embedded in the growth cones of migrating neurons [30,31], where such finger-like processes are thought to sense local cues to guide the migrating neuron to its target [32], whilst the large mesh-like lamellipodium in which they are embedded generates the forces required to drive the growth cone or cell forwards [33-35]. Given this role for *mnb *in shaping actin-based protrusions, we considered two explanations for its neuronal-specific phenotype. First, it is possible that *mnb *is not expressed in hemocyte-derived cells or that the dsRNA failed to silence the *mnb *expression in these cell lines. Q-PCR analysis revealed that *mnb *is expressed and effectively silenced by RNAi in both S2R+ and BG3-c2 cells (Figure 3d), ruling out this explanation. Second, we considered the possibility that the ability to visualize a morphological phenotype associated with the loss of *mnb *was dependent on the shape of the cells used in the analysis. To test whether this might be the case, we forced BG3-c2 cells to spread on a conconavalin A coated substrate (Figure 3b, right-hand panels). Although this led to the formation of broad lamellipodia in control (lacZ RNAi treated) BG3-c2 cells, it was unable to suppress the induction of ectopic filopodia induced by *mnb *depletion. Thus, we cannot attribute the failure of *mnb *dsRNA to elicit an RNAi phenotype in Kc, S2 an S2R+ cells to differences in their form. Instead, these results suggest that *mnb *specifically acts to inhibit the transition between filopodia and lamellipodia in CNS-derived cells. Since *mnb *plays a conserved role in neurogenesis [27], and has a strong cell morphological RNAi phenotype in CNS-derived cell lines, it seems likely that it represents a cell type-specific regulator of cell morphology and behavior.

### Genes with cell type-specific phenotypes are not differentially expressed

**Figure 4:**
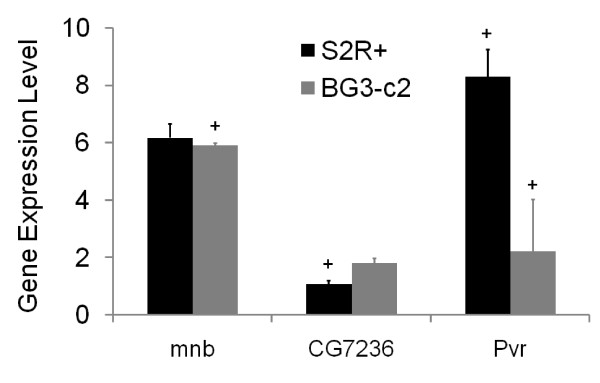
Genes with cell line-specific phenotypes are not differentially expressed. Chart of the expression levels of *CG7236*, *mnb *and *Pvr *in S2R+ and BG3-c2 cells as measured by Q-PCR. Expression levels were established by taking the ratio of expression of each gene compared to the control ribosomal component rp49 in three independent experiments. The error bars represent the standard error in the mean across those experiments. Plus sign indicates RNAi treatments that resulted in an observed phenotype.

In order to test whether these phenotypic differences reflect differences in gene expression between different cell lineages, we used the gene expression analysis to determine whether the differences in gene expression correlate with differences in function, as ascertained using RNAi across the kinome (Additional data files 6 and 7). We were unable to identify such a correlation. However, given the potential problems with a global microarray analysis, we followed this up using Q-PCR to establish whether the relative levels of *mnb *and *CG7236 *expression in S2R+ and BG3-c2 cells correlate with their cell type-specific functions. *Pvr*, a gene that displayed similar strong phenotypes in all cell types screened, was used as a control for this analysis. It was obvious from this analysis that there was no strong correlation between expression at the mRNA level and function (Figure 4; Additional data files 6 and 7). Thus, we identified no clear difference in *mnb *expression levels between neuronal and hemocyte cell lines, and *CG7236 *mRNA levels were lower in S2R+ cells, where RNAi causes a phenotype, than they were in BG3-c2 cells (Figure 4). Furthermore, *Pvr*, which displayed strong phenotypes in both S2R+ and BG3-c2 cells, was expressed at very different levels in the different cell lines (Figure 4). These data suggest that, in the case of the kinases at least, there is no simple relationship between gene expression level and function.

## Discussion

Using parallel RNAi screens in different cell lines, we have identified a new CNS-specific function for *mnb/*DYRK1A, a protein previously shown to play a role within both the fly and mammalian CNS, in the regulation of the structure of actin-based protrusions. This lineage-specific function of Mnb was not determined by cell shape or by *mnb *transcription. Significantly, these data demonstrate the dangers of using data from an RNAi screen carried out in one cell type to make general statements about the function of a gene, and the difficulties of using gene expression as a guide to cell type-specific differences in gene function. Furthermore, when we compared the hits identified in our parallel RNAi screens in neuronal cell lines with those identified in a recently published genome-wide high-content RNAi screen in *Drosophila *primary neurons by Sepp *et al*. [36], there was no overlap. Although there are a number of possible simple explanations for this, we think this is unlikely to reflect differences in the RNAi libraries, given the relatively low false positive (7-18%) and false negative (13-27%) rates in our screen. However, the method used to reveal changes in cell shape was different in each case, with the screen by Sepp *et al*. using CD8-green fluorescent protein to reveal cell morphology, rather than fixing and staining to determine cytoskeletal organization. In addition, the hit detection methods used were different in the two cases. More fundamentally, however, Sepp *et al*. used differentiating primary neurons isolated from stage 6-8 *Drosophila *embryos for their analysis, where maternal loading of protein will have a major impact on the ability of given dsRNA to induce a phenotype, whereas we used stable neuronal cell lines as our model systems, which may not serve as well as models of differentiated neurons. Once again, however, the comparison emphasizes the need for caution when extrapolating RNAi phenotypic data between systems.

## Conclusion

This analysis shows how a functional genomic approach can be used to differentiate between generic and cell type-specific gene functions, and how phenotypic data can be used to cluster cells into groups that are related by origin and morphology. It also reveals the benefits of using multiple non-overlapping dsRNAs to help estimate false positive and false negative rates in such screens. Finally, although the phenotypic groups identified resemble clusters generated using a gene expression array analysis, our study reveals the dangers of using gene expression data to predict function, and in doing so demonstrates the importance of cell type-specific RNAi screening as an approach for dissecting pathways of cellular control.

## Materials and methods

### dsRNA synthesis and kinase library generation

Pairs of gene-specific primers (QIAGEN, West Sussex, UK) were taken from the FLIGHT database [16] or designed *de novo *using the E-RNAi primer design tool [37]. Each primer was designed to be approximately 21 bp in length before addition of a T7 tag. Templates for the kinome RNAi library, targeting 265 *Drosophila *kinases and kinase regulatory subunits (Additional data file 1) were generated by PCR using HotSarTaq DNA polymerase (QIAGEN). dsRNA synthesis was performed using the T7 MegaScript kit (Applied Biosystems, Foster City, California, USA). RNA preparations were purified using a Multiscreen PCR purification kit (Millipore Corporation, Bedford, MA, USA) attached to a vacuum pump. Purified RNAs were annealed by heating at 65°C for 10 minutes and cooling slowly. PCR and dsRNA synthesis were performed in 96-well plates and dsRNA concentrations were adjusted to 1 μg/μl before aliquoting into 384-well assay plates using a Beckman Biomek FX robot (Beckman Coulter (U.K.) Limited, Buckinghamshire, UK).

### Tissue culture

Six *Drosophila *cell lines were used in this study. Kc167 and S2R+ cells were grown in Schneider's medium (Invitrogen, Carlsbad, California, USA) with 10% heat-inactivated fetal bovine serum (JRH Biosciences, Kansas, USA) and 1% penicillin-streptomycin (Sigma-Aldrich, St Louis, Missouri, USA) at 24°C in treated culture flasks (Falcon from BD Biosciences, San Jose, California, USA). S2R+ cells were removed from culture flasks using Trypsin-EDTA (Invitrogen). S2 cells were grown in InsectExpress media with L-Glutamine (PAA Laboratories, Pasching, Austria). The BG2-c2, BG3-c2, and BG3-c1 cell lines were cultured with Shields and Lang M3 insect medium (Sigma) with fetal bovine serum and antibiotics. M3 medium was supplemented with insulin for BG3-c2 (10 μg/ml) and BG3-c1 (5 μg/ml) cells.

### RNAi screening and automated image acquisition

For RNAi screens, cells in serum-free medium were plated into 384-well assay plates containing dsRNA (20 μg/ml final concentration) by the Thermo Scientific Matrix WellMate mutlidrop machine (Thermo Fisher Scientific, Hudson, New Hampshire, USA), centrifuged briefly, then incubated at 24°C for 15 minutes before addition of complete medium. Cells were grown for 5 days at 24°C. In each experiment, positive and negative controls (pebble/thread/SCAR/LacZ RNAi) were included. Cells were fixed for 10 minutes in 4% formaldehyde (Polyscience, Niles, Illinois, USA). After fixation, cells were permeabilized by washing with phosphate-buffered saline (PBS) containing 0.1% Triton-X-100 (PBS-T), then blocked with 5% bovine serum albumin in PBS-T for 15 minutes. Cells were incubated with primary antibody (α-Tubulin) in PBS containing 1% bovine serum albumin overnight at 4°C. Cells were then washed and incubated with secondary antibody (FITC anti-mouse IgG) combined with TRITC-Phalloidin and DAPI for 2 hours. After staining, cells were washed and stored in 0.1% sodium azide in PBS-T at 4°C sealed with Costar6570 Thermowell sealing tape.

Fluorescent images were acquired using an automated Nikon TE2000 microscope with a 20× objective and HTS MetaMorph software (Universal Imaging, Molecular Devices, Downingtown, Pennsylvania, USA) running an automated stage, filter wheel and shutter, and a cooled-coupled device camera (Hamamatsu, Nishi Ward, Hamamatsu City, Japan). Automated wide focusing was performed on the DAPI channel first. Images were acquired in three channels at three sites per well. All image data and annotations are available online through the FLIGHT database [16].

### Two step reverse transcriptase Q-PCR

Cytoplasmic RNA was harvested from BG3-c2 and S2R+ cells using the RNeasy miniprep kit (Qiagen) according to the manufacturer's guidelines. SuperScript II Reverse Transcriptase kit (Invitrogen) was used to synthesize the first-strand cDNA according to the manufacturer's guidelines. *Escherichia coli *RNase H was used to remove RNA complementary to the cDNA. Q-PCR was performed using SYBR green (Invitrogen Molecular Probes) and an MX4000 real-time PCR machine (Stratagene, La Jolla, California, USA). SYBR green fluorescence was quantified using a serial dilution of template containing PCR products of known concentration. Relative abundance of transcript was normalized against control (rp49) RNA levels. Primer sequences (Eurogentec, Southampton, Hampshire, UK) for all genes can be found in Additional data file 8.

### Cell number measurement

Cell number counts were used to gain a quantitative assessment of the *mnb *phenotype in S2, BG2, BG3-c1 and BG3-c2 cells. In each case, 1.5 × 10^6 ^cells were treated with *mnb *or *lacZ *dsRNA in a 4-well-dish. On the fifth day, cells were counted in triplicate using a Beckman Z2 Coulter Counter. The average cell number and standard deviation are presented for each.

### Microarray gene expression analysis

Total mRNA from wild-type cells in exponential growth phase was isolated by TRIzol extraction (Invitrogen). Microarray gene expression analysis was carried out using FlyChip long oligonucleotide spotted microarrays (FL002). Expression data were Loess normalized by intensity and probe location per chip, and rank normalized across chips. Normalized expression was then averaged across three replicate chips for each cell line. Hierarchical clustering was performed using the Pearson correlation and the average linkage method. All data processing was performed using R/Bioconductor [38]. All gene expression data are available online from the FLIGHT database [16].

## Abbreviations

CNS: central nervous system; dsRNA: double-stranded RNA; DYRK: dual specificity tyrosine-phosphorylation-regulated kinase; PBS: phosphate-buffered saline; Q-PCR: quantitative PCR; RNAi: RNA interference.

## Authors' contributions

TL carried out the RNAi screens, cell biology, and the Q-PCR. DS performed the microarray studies and the computational analysis of RNAi screen results. BB conceived of the study, and participated in its design and coordination. TL, DS and BB drafted the manuscript.

## Additional data files

The following additional data are available with the online version of this paper: details of the primer sequences used to generate the *Drosophila *kinase RNAi library and to estimate false positive and false negative rates in the screen (Additional data file 1); estimates of screen false positive and negative rates (Additional data file 2); details of the kinases that were found to be cell morphology hits in each of the six different *Drosophila *cell lines (Additional data file 3); a figure showing Mnb phenotypes in BG2-c2 and BG3-c1 cell lines (Additional data file 4); a figure showing the effect of *mnb *RNAi on cell number in CNS-derived cell lines (Additional data file 5); a figure showing a comparison of microarray gene expression levels of genes displaying phenotypes in S2R+ and BG3-c2 cells (Additional data file 6); a figure showing a summary of the gene expression of all genes with phenotypes across all cell lines (Additional data file 7); details of the primers used for Q-PCR (Additional data file 8).

## Supplementary Material

Additional data file 1 Primer sequences used to generate the *Drosophila *kinase RNAi library and to estimate false positive and false negative rates in the screen. Click here for file

Additional data file 2 This file contains details of the false positive and false negative analysis performed in this study and the effect of dsRNA quality on false negatives. Click here for file

Additional data file 3 Kinases found to be cell morphology hits in each of the six different *Drosophila *cell lines. Click here for file

Additional data file 4 Actin staining shows similar phenotypes with that of BG3-c2 for *mnb *RNAi in BG2-c2 and BG3-c1 cell lines. Click here for file

Additional data file 5 Silencing of *mnb *expression by RNAi causes an average 25% reduction of cell numbers in BG3-c1, BG3-c2 and BG2-c2 cell lines, but has no effect in S2 cells, four days after dsRNA treatment. Click here for file

Additional data file 6 Chart of the gene expression levels determined in the microarray analysis for genes showing phenotypes in both S2R+ and BG3-c2 cells compared to those with phenotypes in BG3-c2 or S2R+ cells. There is no strong pattern of gene expression associated with genes with cell type-specific phenotypes. Click here for file

Additional data file 7 Pie chart summarizing the gene expression profiles (present or absent) of genes showing phenotypes in any cell line. The vast majority of genes with expression data available are either present in all cell lines tested, or absent from all. This suggests that cell type specific phenotypes do not arise simply from expression of different subsets of signaling components. Click here for file

Additional data file 8 Primers used for Q-PCR. Click here for file
