# Supplementary figures and images for: Parallel RNAi screens across different cell lines identify generic and cell type-specific regulators of actin organization and cell morphology
Source: Genome Biol. 2009 Mar 5;10(3):R26. doi: 10.1186/gb-2009-10-3-r26 (PMC2690997; doi:10.1186/gb-2009-10-3-r26)

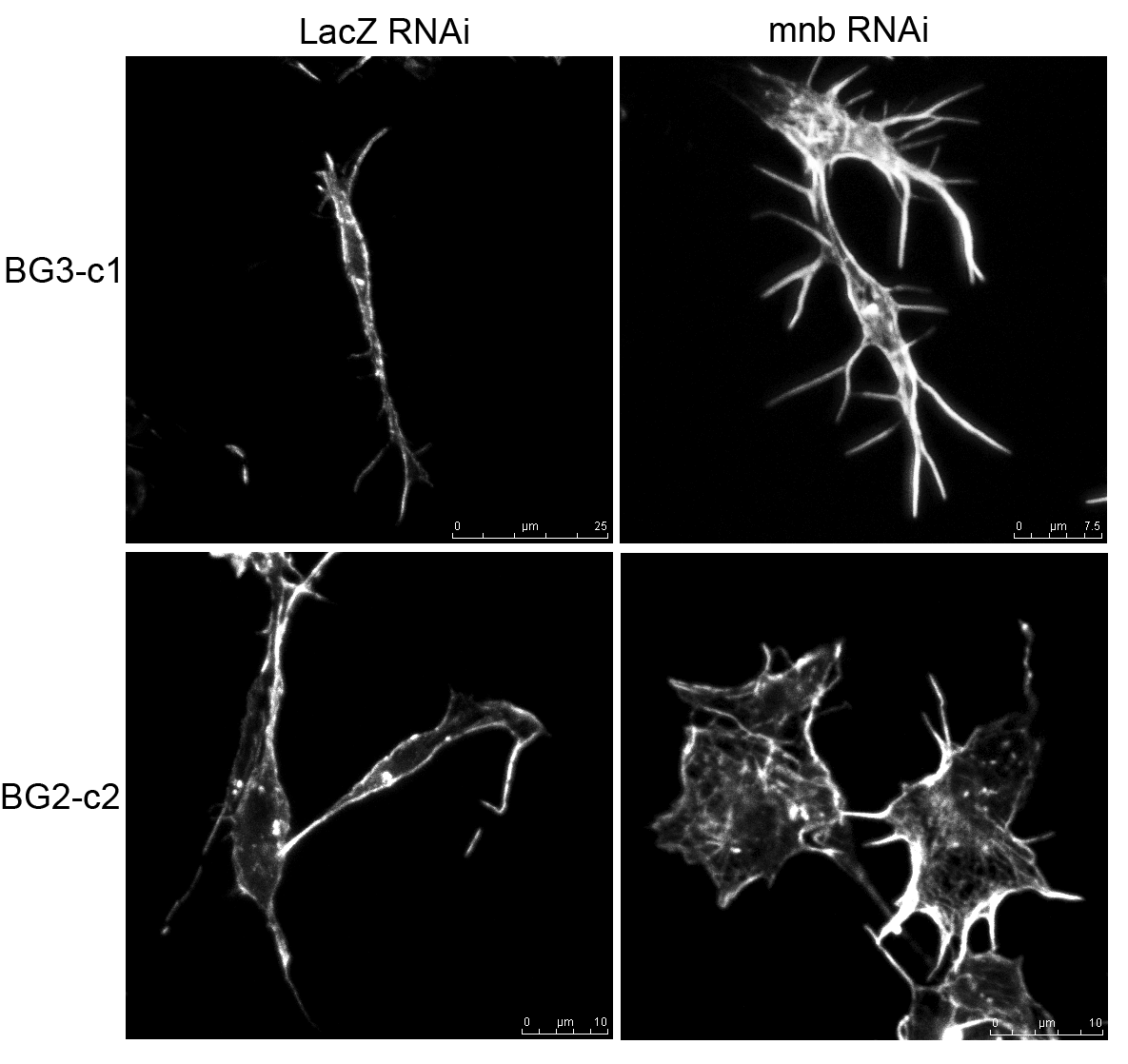

Supplement: Additional data file 4 — Actin staining shows similar phenotypes with that of BG3-c2 for mnb RNAi in BG2-c2 and BG3-c1 cell lines. [file gb-2009-10-3-r26-S4.tiff]

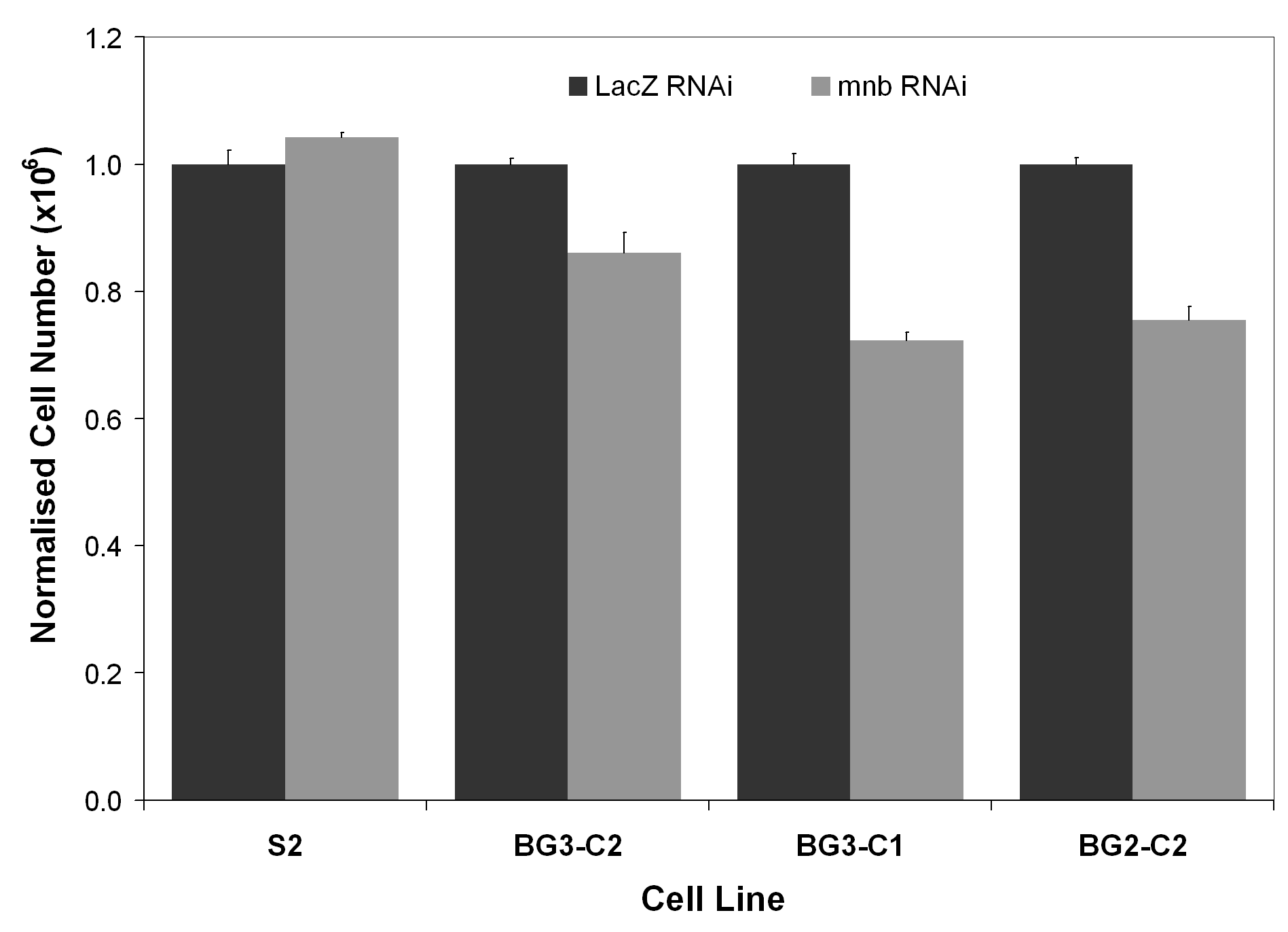

Supplement: Additional data file 5 — Silencing of mnb expression by RNAi causes an average 25% reduction of cell numbers in BG3-c1, BG3-c2 and BG2-c2 cell lines, but has no effect in S2 cells, four days after dsRNA treatment. [file gb-2009-10-3-r26-S5.tiff]

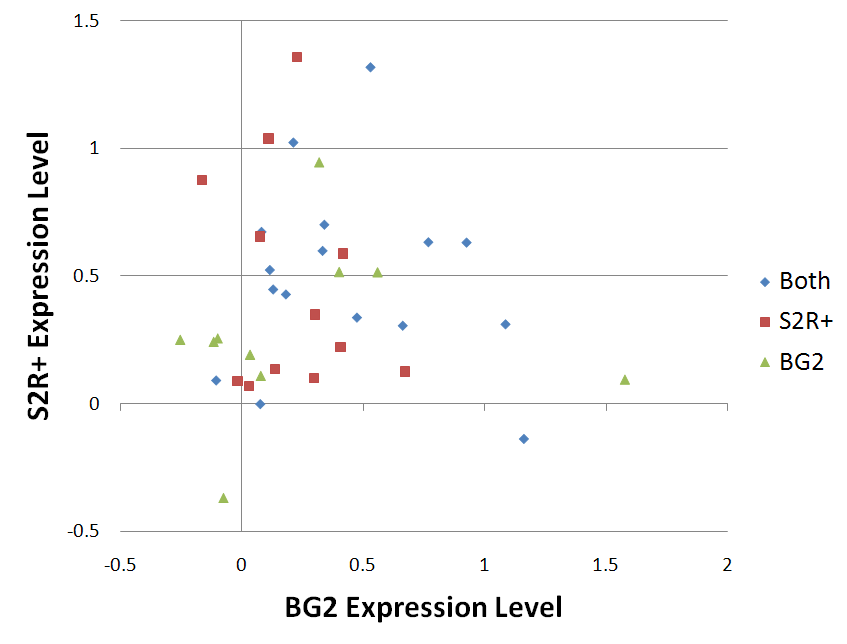

Supplement: Additional data file 6 — Chart of the gene expression levels determined in the microarray analysis for genes showing phenotypes in both S2R+ and BG3-c2 cells compared to those with phenotypes in BG3-c2 or S2R+ cells. There is no strong pattern of gene expression associated with genes with cell type-specific phenotypes. [file gb-2009-10-3-r26-S6.tiff]

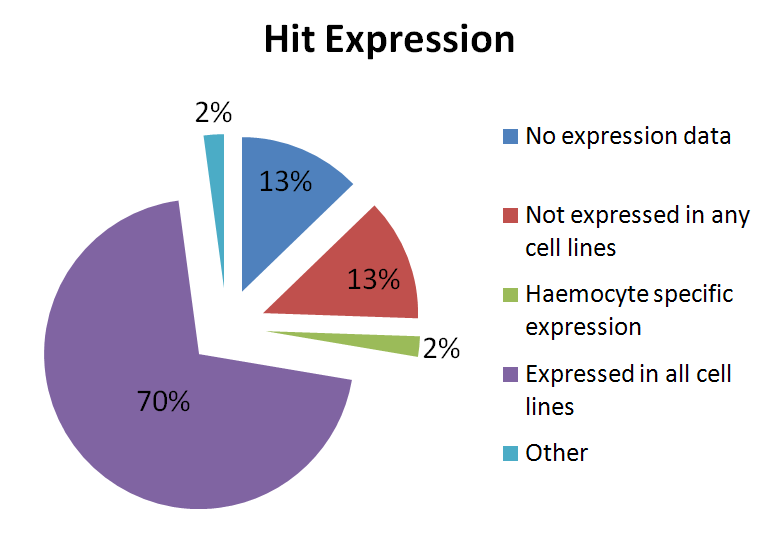

Supplement: Additional data file 7 — Pie chart summarizing the gene expression profiles (present or absent) of genes showing phenotypes in any cell line. The vast majority of genes with expression data available are either present in all cell lines tested, or absent from all. This suggests that cell type specific phenotypes do not arise simply from expression of different subsets of signaling components. [file gb-2009-10-3-r26-S7.tiff]
